# Case report: successful resection of a leiomyoma causing pseudoachalasia at the esophagogastric junction by tunnel endoscopy

**DOI:** 10.1186/s12876-016-0445-0

**Published:** 2016-02-25

**Authors:** Bin Deng, Xue-Feng Gao, Yun-Yun Sun, Yuan-Zhi Wang, Da-Cheng Wu, Wei-Ming Xiao, Jian Wu, Yan-Bing Ding

**Affiliations:** Department of Gastroenterology, the Second Clinical College of Yangzhou University, Yangzhou, Jiangsu 225001 China; Department of Gastroenterology, Yangzhou No. 1 People’s Hospital, 368# of HanJiang middle road, Yangzhou, Jiangsu 225001 China

**Keywords:** Pseudoachalasia, Esophagogastric junction, Leiomyoma

## Abstract

**Background:**

Pseudoachalasia is a rare disorder whose presentation strongly resembles idiopathic achalasia.

**Case presentation:**

Here, we present a case of a 42-year-old female patient with esophageal leiomyoma who was initially diagnosed with achalasia. On endoscopical investigation, however, it became apparent that she had pseudoachalasia as consequence of a leiomyoma at the esophagogastric junction (EGJ). The condition was successfully treated through submucosal tunneling endoscopic resection.

**Conclusion:**

This case suggests that submucosal tunneling endoscopic resection is a therapeutic u option for the treatment of pseudoachalasia caused by leiomyoma of EGJ.

## Background

Achalasia is the most common functional gastrointestinal disorder. It is characterized by aberrant upper gastrointestinal motility due to loss of peristalsis in the lower two-thirds of the esophagus and impaired relaxation of the lower esophageal sphincter [1, 2]. Achalasia can sometimes be difficult to distinguish from pseudoachalasia, a term that covers a spectrum of disorders that can mimic the clinical and investigational presentation of idiopathic achalasia [3]. In the herein presented case, general clinical assessment as well as endoscopic, manometric, and radiologic studies suggested a diagnosis of achalasia, and as a consequence the patient was scheduled to undergo peroral endoscopic myotomy (POEM). However, on performing the endoscopy for POEM, a leiomyoma originating from the muscularis propria (MP) layer and located at the esophagogastric junction was found. A revised diagnosis of pseudoachalasia was made. As a consequence the patient was subsequently treated with submucosal tunneling endoscopic resection (STER).

## Case presentation

**Fig. 1:**
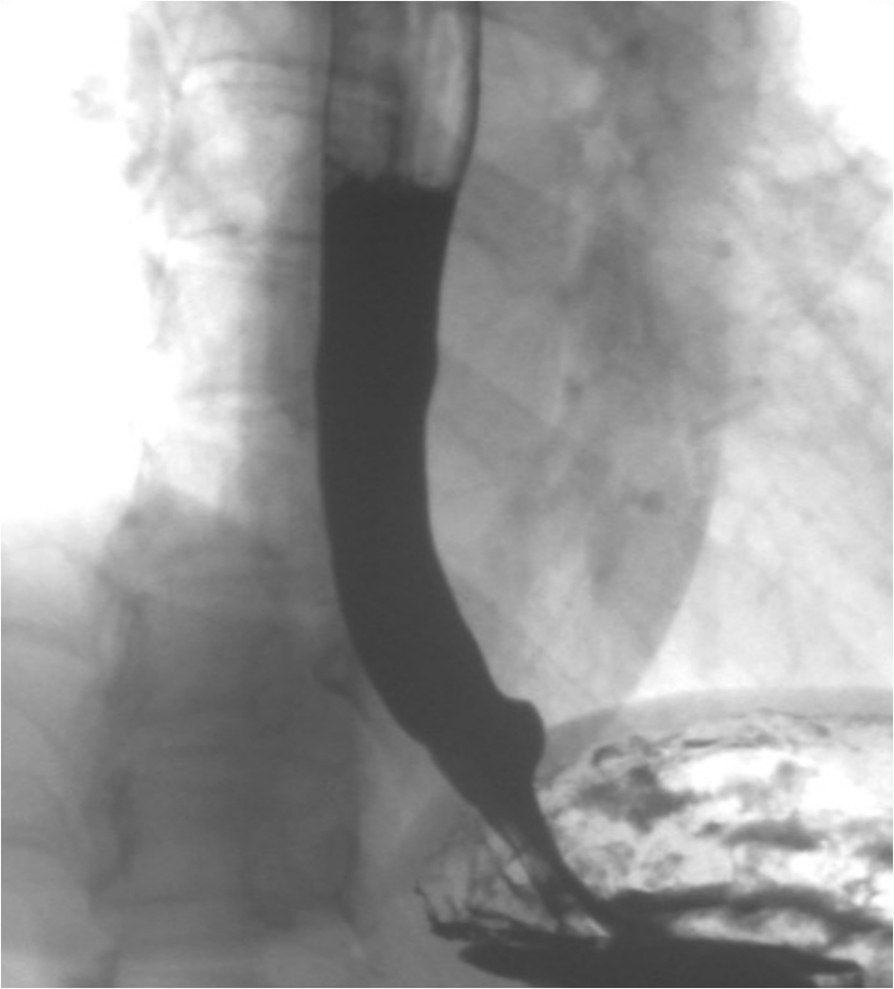
Radiograph of the barium esophageal transit examination. The findings have characteristic appearance of achalasia

**Fig. 2:**
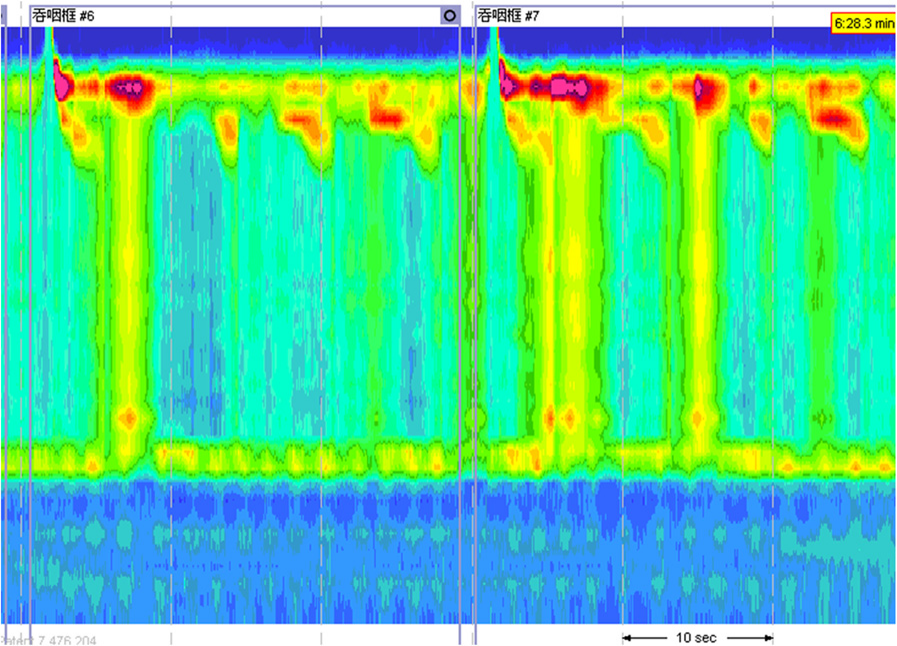
Esophageal high-resolution manometry of our patient. The results show that lower esophageal sphincter pressure is reduced, with concomitant relaxation and passage of lower esophageal sphincter seen upon swallowing. The results are concordant with achalasia type II

**Fig. 3:**
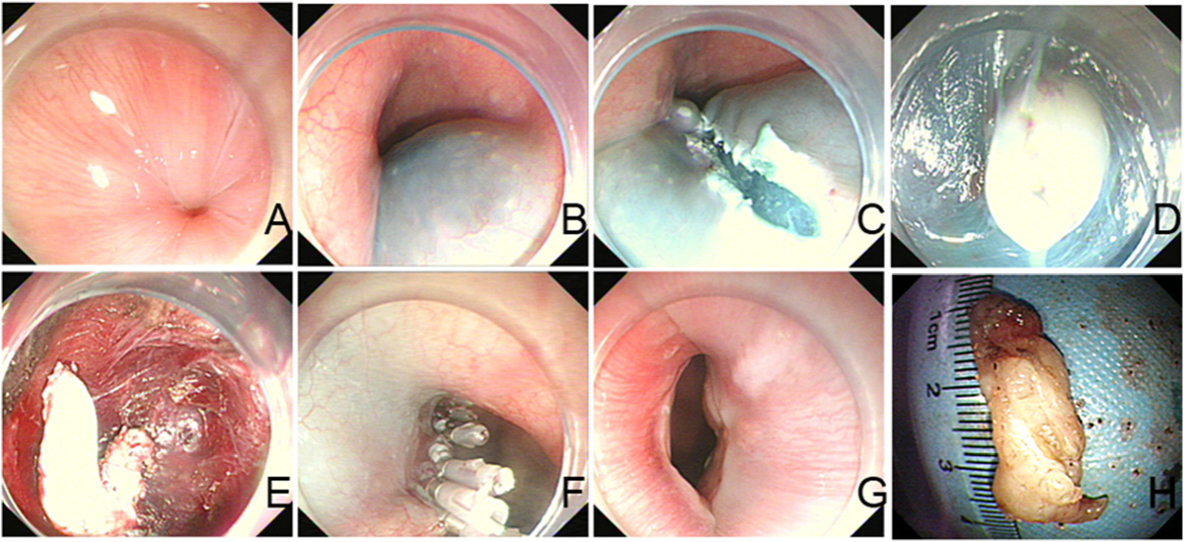
Illustration of the endoscopic technique. **a** Endoscopic view of esophagogastric junction before endoscopic therapy. **b** Submucosal injection of diluted indigo carmine. **c** A 2-cm longitudinal mucosal incision was made approximately 10 cm proximal to the gastroesophageal junction. **d** Appearance of the exposed tumor in the submucosal tunnel. **e** Impression of the endoscopic view during the tumor resection. **f** Mucosal entry incision sealed with several clips. **g** Endoscopic view of the esophagogastric junction after endoscopic therapy. **h** Resected tumor specimen measuring 3.0 cm

**Fig. 4:**
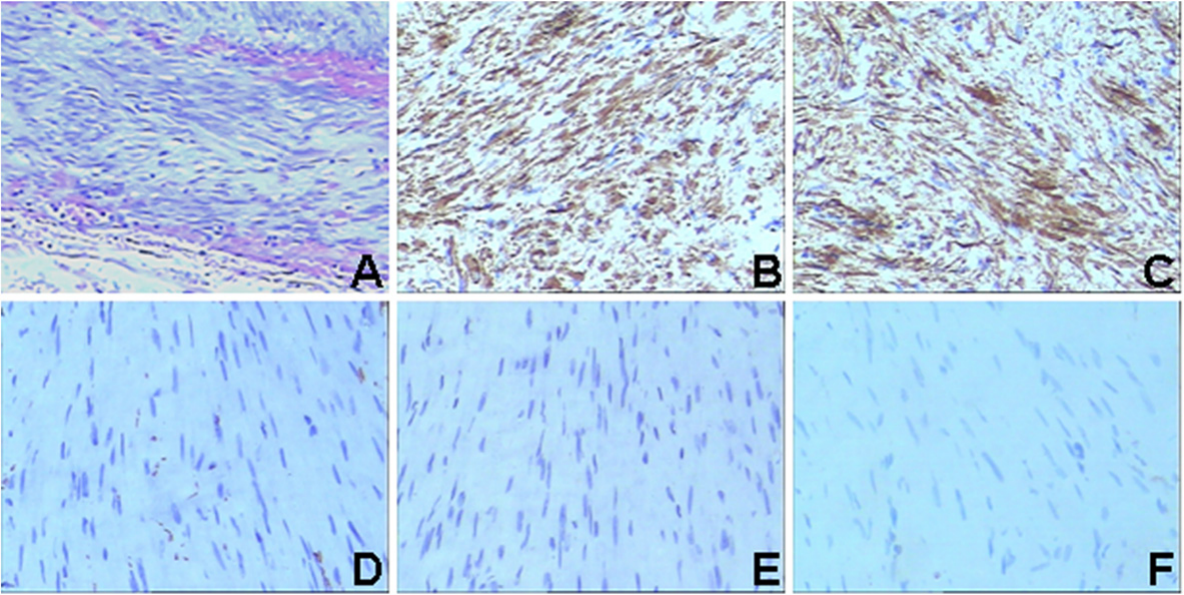
Histological evaluation reveals the typical morphology of leiomyoma. Histopathological changes of the tissues determined by H&E staining (**a**), Expression of SMA (**b**), Desmin (**c**), CD34 (**d**), CD117 (**e**), and Dog-1 (**f**) as assessed by immunohistochemistry. Magnification × 200. Also note the apparently complete resection achieved

A 42-year-old female patient was referred to the Department of Gastroenterology at the Second Clinical College of Yangzhou University, for long-standing complaints of solid and liquid dysphagia accompanied by episodes of regurgitation. She underwent two upper digestive endoscopies, on which the presence of food residues in the esophageal lumen and rebound passage through the gastroesophageal junction was observed. Gastroesophageal contrast radiography showed slight dilatation of the esophagus, an absence of primary peristalsis but presence of aperistaltic waves, and a narrow distal esophagus with a ‘bird’s beak’ aspect (Fig. 1). A CT scan was performed, but was unremarkable and did not reveal any esophageal lesion. Esophageal manometry demonstrated a hypertensive lower esophageal sphincter pressure in conjunction with incomplete relaxation and isobaric aperistaltic pressurizations, all consistent with type II achalasia (Fig. 2). The patient was counseled regarding the diagnosis of achalasia and scheduled for POEM. Informed patient consent was obtained before the procedure. The patient was intubated and brought under general anesthesia. Subsequently, upper digestive endoscopy was performed by using a conventional endoscope. Carbon dioxide insufflation was used throughout the procedure. At the esophageal mucosa, 10 cm proximal to the gastroesophageal junction, a 2 cm longitudinal mucosal incision was made using a Hook knife to serve as the entry point for the tunnel in the planned POEM procedure. The mucosa was then separated from muscular layer starting at beginning of the tunnel and the endoscope was then used to enlarge the submucosal tunnel by further separation of mucosa and muscularis. When the channel reached the proximity of the esophagogastric junction, however, a white band of potential tumor tissue became apparent. On encountering this structure, it was decided to resect the putative tumor by separating it from the surrounding muscular layer under direct endoscopic view using an insulated-tip knife. The then mobilized tumor was excised out of the tunnel using a snare through the mucosal entry. The endoscope was then withdrawn from the submucosal tunnel and was capable of passing the gastroesophageal junction without apparent difficulty, thus the myotomy was aborted. Several metal clips were employed to close the mucosal incision (Fig. 3). Subsequent histological evaluation combined with relevant immunohistochemistry produced a definitive diagnosis of leiomyoma (Fig. 4). Now, 10 months postoperatively the patient is largely asymptomatic.

## Conclusions

Achalasia is a primary esophageal motility disorder, which manifests itself through dysphagia to liquids and solid foods. Support for a diagnosis of achalasia can be obtained by endoscopic and radiological studies, but the mainstay of the diagnostic process consists of the manometric findings. Classically, three characteristic manometric features are present in achalasia: an elevated resting lower esophageal sphincter pressure, incomplete lower esophageal sphincter relaxation, and esophageal aperistalsis [4]. Pseudoachalasia is characterized by achalasia-like symptoms caused by secondary etiologies. Clinical, radiological, and endoscopic findings closely resemble those of achalasia, but the treatment and associated prognosis are markedly different [5]. Hence, correct discrimination between achalasia and pseudoachalasia is important, but it is frequently complicated by the absence of specific pseudoachalasia-specific symptoms or findings and the relative rarity of pseudoachalasia. In our patient, the overall clinical, endoscopic, and radiographic findings clearly favored achalasia, especially in view of the concordant manometric classic type II achalasia pattern observed. Why the pseudoachalasia presented with this manometry remains unresolved and highlights the challenges that could be encountered when trying to make the correct diagnosis in this context. However, the observation of the lower esophageal leiomyoma made a differential diagnosis of pseudoachalasia more likely. Leiomyoma is the main type of submucosal tumor (SMTs) in the esophagus and its presence may manifests itself in dysphagia [6]. Interestingly, achalasia misdiagnosis in SMT-mediated dysphagia has been reported multiple times in previous studies [7, 8] and thus this alternate diagnosis should always be in the differential diagnosis even when achalasia-concordant manometry is available. Moreover, clues to distinguish achalasia from pseudoachalasia can result from endoscopic ultrasound (EUS) [9], thus this case supports the use of EUS investigation upon this type of clinical presentation. The present case management did not involve EUS before endoscopic surgery, but in retrospect this would have been desirable.

POEM is a novel endoscopic technique for the treatment of achalasia. Since Inoue et al. [10] reported its safety and effectiveness in humans, many groups have reported similar results with minimal complications [11, 12]. Based on POEM, Xu et al. [13] developed a new endoscopic technique named STER for treating SMTS originating from the muscularis propria in the esophagus. It has already been reported that STER is also safe, effective, and feasible with respect to SMTs at the esophagogastric junction, combining the possibility for accurate histopathologic evaluation of the lesion with its curative treatment [14]. The successful application of STER in the present study supports this notion. As both POEM and STER are in essence endoscopic tunneling techniques, they share most of technical procedures. For the present case, this was a fortunate coincidence since POEM was initiated while STER was needed to be performed, and thus secondary surgery could be avoided. It is clear, however, from this case and the body of available literature that STER might be an option for the treatment of pseudoachalasia caused by leiomyoma of EGJ.

## Consent

Written informed consent was obtained from the patient for publication of this case report and any accompanying images. A copy of the written consent is available for review by the Editor in Chief of this journal. This study was reviewed and approved by the Institutional Review Board of Second Clinical College of Yangzhou University.

## Abbreviations

EGJ: Esophagogastric junction
POEM: Peroral endoscopic myotomy
MP: Muscularis propria
STER: Submucosal tunneling endoscopic resection
SMT: Submucosal tumor
EUS: Endoscopic ultrasound
